# Injecting hemostatic matrix in the path of biopsies: efficacy, potential complications, and the management of such complications

**DOI:** 10.1590/0100-3984.2017.0011

**Published:** 2018

**Authors:** Antonio Rahal Junior, Priscila Mina Falsarella, Vinicius Tadeu Rodrigues Ferreira, Guilherme Cayres Mariotti, Marcos Roberto Gomes de Queiroz, Rodrigo Gobbo Garcia

**Affiliations:** 1 MD, Physician in the Department of Interventional Radiology of the Hospital Israelita Albert Einstein, São Paulo, SP, Brazil.; 2 Researcher in the Department of Interventional Radiology of the Hospital Israelita Albert Einstein, São Paulo, SP, Brazil.

**Keywords:** Hemostatic techniques, Hemostatics/administration & dosage, Biopsy, needle/methods, Hemorrhage/prevention & control, Técnicas hemostáticas, Hemostáticos/administração & dosagem, Biópsia por agulha/métodos, Hemorragia/prevenção & controle

## Abstract

**Objective:**

To describe the technique of injecting hemostatic matrix, as well as the experience of our interventional radiology department in its application.

**Materials and Methods:**

We conducted a single-center study with retrospective analysis of the experience of our group in the use of hemostatic gelatin matrix in percutaneous biopsies.

**Results:**

In a total of 73 biopsies in different organs, such as the liver, kidney, and spleen, hemostatic gelatin matrix was introduced into the coaxial needle. The only complication observed was migration of the hemostatic matrix to the left kidney collecting system, and that was resolved with clinical treatment. There were no cases of bleeding after the injection of hemostatic matrix.

**Conclusion:**

The use of hemostatic matrices in the path of percutaneous biopsies is another tool available for consideration in minimally invasive procedures.

## Abstract (pt)

**Objetivo:**

Descrever a técnica de injeção de matrizes hemostáticas e a experiência do nosso serviço de radiologia intervencionista na sua aplicação.

**Materiais e Métodos:**

Foi realizado estudo unicêntrico com análise retrospectiva da experiência do nosso grupo na utilização de matriz hemostática gelatinosa em biópsias percutâneas.

**Resultados:**

Foram realizadas 73 biópsias com utilização de matriz hemostática gelatinosa no trajeto da agulha coaxial em diferentes órgãos, como fígado, rim, baço, entre outros. A única complicação observada foi a migração da matriz hemostática para o sistema coletor do rim esquerdo, sendo solucionada com tratamento clínico. Não foram observados casos de sangramento no trajeto das biópsias após a injeção de matrizes hemostáticas.

**Conclusão:**

O uso de matrizes hemostáticas no trajeto de biópsias percutâneas é mais uma ferramenta disponível a ser considerada nos procedimentos minimamente invasivos.

## INTRODUCTION

Unlike conventional surgical techniques, percutaneous biopsies are safe, minimally invasive procedures that are used for the histological diagnosis of solid-organ lesions^(1)^. Despite being safe, such procedures can occasionally provoke bleeding that is difficult to control, especially in patients with coagulopathy^(2)^. Nevertheless, failure to perform a percutaneous biopsy can delay the initiation of the appropriate treatment.

The risk of bleeding from a biopsy depends on several factors, which should be thoroughly evaluated before the procedure^(2)^. The factor that contributes most to the increased risk of bleeding is coagulopathy^(3)^, and the patients who present an increased risk for bleeding are those with a serum platelet count below 50,000/mm^3^ or an international normalized ratio above 1.5. In contrast, certain factors contribute to reducing the risk of bleeding during percutaneous biopsy, such as the transfusion of blood products, if indicated, and the suspension of antiplatelet or anticoagulant agents when possible. Among the technical factors, planning the best access to the lesion and choosing the best imaging method to guide the procedure-be it ultrasound, tomography, or the fusion of ultrasound images with images obtained via other techniques such as magnetic resonance, tomography, and positron emission tomography-collaborate to reduce the risk of bleeding and increase the precision of the technique. The use of a coaxial needle as a guide for the cutting needle is also an important element that reduces the risk of bleeding, because it avoids the repetitive cutting/piercing trauma along the path of the biopsy^(4)^, as is the use of a semiautomatic cutting needle, because it allows greater control in the progression of the tip.

A complementary form of increasing the safety of percutaneous biopsy, by reducing the risk of bleeding, is embolization of the needle path with hemostatic matrices. Although hemostatic matrices have been used in open surgical procedures for decades^(5)^, their use in imaging-guided percutaneous biopsy is a relatively recent application. The hemostatic matrices currently available are composed of various substances, including gelatin, collagen, and cellulose. The most widely used hemostatic matrix is an absorbable gelatin sponge^(2)^. Many of the patients who require biopsy have hemorrhagic disorders^(6)^, such as those with liver or kidney disease^(7)^, and a large part of focal lesions present hypervascularity, which increases the risk of bleeding. Therefore, knowledge and the correct application of this percutaneous biopsy technique, in select cases, has been of great value in medical practice, with a special focus on intervention.

The objective of this study was to describe the technique of injecting hemostatic matrices, as well as the experience of our facility in its application. We also evaluate the associated complications and the management of such complications by interventional radiologists.

## MATERIALS AND METHODS

This was a single-center study in which we performed a retrospective analysis of the experience of our group in the use of a hemostatic matrix based on purified pork skin gelatin (Gelfoam^®^ absorbable gelatin sponge; Pfizer, New York, NY, USA) in percutaneous biopsies. All procedures were carried out in the interventional radiology center of our institution.

### Patients

We reviewed the cases of 73 patients (39 men and 34 women), 12-84 years of age (mean age, 54.1 years; median age, 54 years), who had been referred to the interventional radiology department of our hospital and had undergone imaging-guided percutaneous biopsy with injection of the gelatin sponge in the path of the biopsy, between October 2013 and May 2016.

The inclusion criteria were being at an increased risk of bleeding due to thrombocytopenia or coagulopathy (platelet count below 75,000/mm³ or international normalized ratio above 1.5) and having any associated comorbidity that carries an unacceptable risk of bleeding. We excluded patients with a known allergy to porcine collagen, in whom the use of the gelatin sponge would be contraindicated. All patients had given written informed consent prior to undergoing the biopsy procedure.

### Procedure

After the best route to avoid major structures and vessels had been planned, percutaneous biopsies were performed with an aseptic technique. The biopsies were guided by computed tomography (Somatom Definition AS 40-slice; Siemens, Berlin, Germany) or by ultrasound (iU 22; Philips Healthcare, Andover, MA, USA-Aplio 500 Platinum; Toshiba American Medical Systems, Tustin, CA, USA-or Logiq E9 VNav; General Electric Healthcare, Milwaukee, WI, USA), with or without image fusion. Every biopsy was performed by one of ten experienced interventional radiologists (each with more than five years of experience). The anesthesia used (local anesthesia, with or without sedation, or general anesthesia accompanied by infiltration with a local anesthetic) varied according to the characteristics of the site to be biopsied, the positioning required, and the characteristics of the patient. The procedures were performed with a 16-, 17-, or 19-gauge coaxial needle, depending on the target structure, together with a semiautomatic 18- or 20-gauge cutting needle.

### Technique

**Figure 1:**
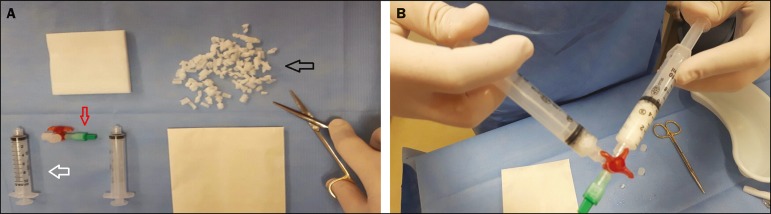
**A:** Gelatin sponge cut into small pieces (black arrow), syringes (white arrow), and three-way stopcock (red arrow) for preparation of the hemostatic matrix paste. **B:** Dilution of the gelatin matrix in saline solution.

After the biopsy, two 10-mL syringes coupled to a three-way stopcock were used in order to mix the gelatin sponge (cut into small pieces of approximately 2 to 3 mm or powdered) into saline until it formed a paste (Figure 1). In each case, 2-4 mL of the paste was administered through the coaxial needle, along the path of the biopsy, from the target site to the organ capsule, a procedure that was also guided by imaging.

After the biopsy, the patients remained under observation during the postanesthesia recovery period. In the first hour, they underwent ultrasound and remained under observation for a period of 4-8 h, depending on the primary site of biopsy (8 h for a random biopsy of a primitive kidney or nodule, and 4 h for biopsies of other structures), with monitoring of vital signs (blood pressure and heart rate), as well as evaluation of the pain score and symptoms. After recovery, the patients were allowed to go home or were transferred to a hospital ward (in the case of previously hospitalized patients).

## RESULTS

A total of 73 biopsies involved the use of the gelatin matrix paste along the path of the coaxial needle. Among those, there were 55 liver biopsies (50 biopsies of focal lesions and 5 random biopsies in liver transplant recipients); 13 kidney biopsies (8 biopsies of primitive kidney biopsies, 4 biopsies of focal lesions, and 1 random biopsy of a transplanted kidney); 1 biopsy of a scapular tumor; 1 biopsy of a perihepatic lymph node; 1 biopsy of an abdominal mass; 1 biopsy of the spleen; and 1 biopsy of a mandibular lesion.

**Figure 2:**
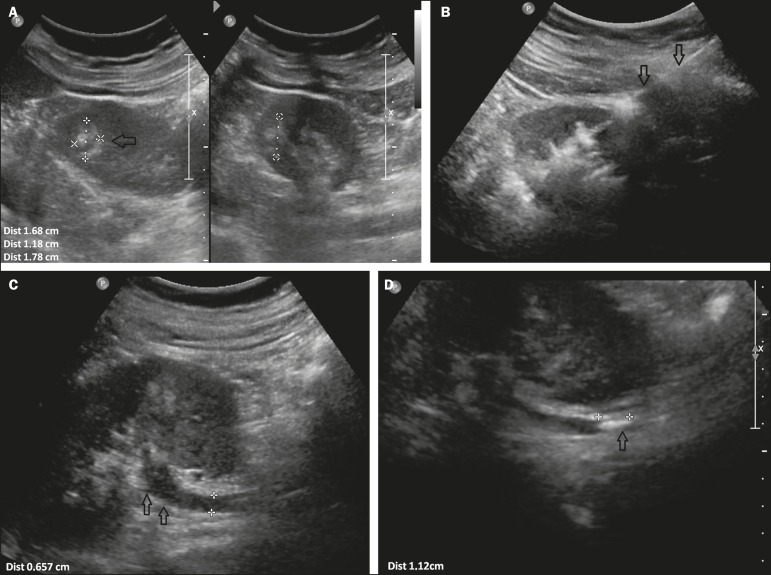
**A:** Renal ultrasound showing a nodule (arrow) in the middle third of the left kidney. **B:** Injection of hemostatic matrix through a coaxial needle (arrows) after the biopsy. **C:** Ureteral dilatation (arrow). **D:** Hyperechoic structure (arrow) in the ureter, accompanied by proximal dilatation.

The only complication observed was migration of the hemostatic matrix to the left collecting system after the biopsy of a central renal lesion, causing obstruction of the distal ureter at the ureterovesical junction and dilatation of the upstream collecting system. That complication was resolved through the use of a three-way indwelling urinary catheter and continuous irrigation with saline solution (Figure 2). There were no instances of bleeding along the path of the biopsies after the injection of the hemostatic matrices, and there was therefore no need for blood transfusion or other therapies.

## DISCUSSION

Bleeding is the most common and one of the most feared complications of solid-organ biopsy^(1)^. The use of hemostatic matrices has been an important tool in these minimally invasive procedures, especially in patients with coagulopathy or hypervascular focal lesions. Its application has been shown to be effective in biopsies of various organs, including the kidney, liver, and other parenchymal organs, as well as of solid masses.

The gelatin sponge has the advantages of being absorbable in days to weeks (depending on the quantity used), easily adapting to the site of application, and absorbing up to 40 times its weight in blood or fluids^(2)^. The main observed benefit of injecting the gelatin sponge along the path of a biopsy in patients with coagulopathies is the reduction of the risk of bleeding due to the tamponade exerted by expansion of the matrix at the site of application, where it forms an artificial clot and facilitates coagulation^(8)^. However, it also has certain risks, including the possibility of migration, due to low tissue adhesion, the formation of a granuloma or local abscess, compressive symptoms resulting from matrix expansion, and allergies^(9-11)^. The disadvantages of the procedure are due to the low tackiness of the solution, and the suspension can be eliminated into the bloodstream in areas with active bleeding^(2)^.

In a study of percutaneous hepatic biopsies performed in dogs, Paulson et al.^(12)^ demonstrated an immediate reduction in the bleeding rate when a hemostatic matrix was introduced in the path of the biopsy course, the rate being lower than that observed in the control group, as well as when anticoagulation therapy was administered, with or without the use of the hemostatic matrix.

It is noteworthy that, despite the advantages of this technique, its application is justified primarily in patients at high risk for bleeding, either due to the type of lesion treated or due to coagulopathies such as those presented by patients with chronic kidney disease, liver transplant recipients, and the elderly, because of the potential for multiple associated comorbidities. The data obtained from our sample are in agreement with those of other studies in the literature^(6,13)^.

Our study has some limitations, including its retrospective design and lack of a control group for comparison. In addition, there was considerable variability in the sites of application and needle sizes used.

## CONCLUSION

The injection of hemostatic matrices in the path of percutaneous biopsies is yet another readily available tool to be considered in minimally invasive procedures. The use of the technique can reduce the risk of bleeding, especially in the most critical patients.
